# Caffeine delays oocyte aging and maintains the quality of aged oocytes safely in mouse

**DOI:** 10.18632/oncotarget.15292

**Published:** 2017-02-11

**Authors:** Xia Zhang, Xiaoyan Liu, Li Chen, Dan-Ya Wu, Zheng-Wen Nie, Ying-Ying Gao, Yi-Liang Miao

**Affiliations:** ^1^ Institute of Stem Cell and Regenerative Biology, College of Animal Science and Veterinary Medicine, Huazhong Agricultural University, Wuhan, China; ^2^ Key Lab of Agricultural Animal Genetics, Breeding, and Reproduction of Ministry of Education, Huazhong Agricultural University, Wuhan, China; ^3^ The Cooperative Innovation Center for Sustainable Pig Production, Wuhan, China; ^4^ Reproductive Medicine Centre, Affiliated Hospital of Qingdao Medical University, Yuhuangding Hospital of Yantai, Yantai, Shandong, China

**Keywords:** oocyte aging, caffeine, mouse, oocyte quality, Gerotarget

## Abstract

Caffeine, as an oocyte aging inhibitor, was used in many different species to control or delay oocyte aging. However, the safety of caffeine and developmental competence of aged oocytes inhibited by caffeine has not been studied systematically. So we detected the spindle morphology, distribution of cortical granules, zona pellucida hardening and pronucleus formation to assess oocyte quality of caffeine treated oocytes. We found that aged oocytes treated by caffeine maintained weak susceptibility to activating stimuli and regained normal competent after aged further 6 hr. Caffeine maintained the spindle morphology, changed cortical granules distribution of aged oocytes and could not prevent zona pellucida hardening. Furthermore, caffeine increased pronucleus formation of aged oocytes and decreased fragmentation after fertilization. These results suggested that caffeine could maintain the quality of aged oocytes safely in mouse.

## INTRODUCTION

The quality of oocyte influences the embryo's developmental potential after fertilization [1]. It has been proved that the optimal window for fertilization in mouse was 8-12 hr after ovulation. Oocyte became aged and the quality of oocyte would decrease if fertilization did not finish during this window [2]. Fertilized oocytes from B6D2F1 and ICR mouse lost their full-term developmental potential by 14 hr and 18 hr after ovulation [3]. An intact meiotic spindle is critically important for accurate distribution of chromosomes to the dividing blastomeres, thus ensuring accurate embryo development. The spindles in aged oocytes became smaller and could be bi- or multipolar, which would block chromosome segregation and result in abnormalities [2]. The main reason was that oocyte aging induced a loss of centrosome structure at the meiotic poles, which was associated with loss of microtubule integrity and chromosome maintenance at the metaphase plate. Oocyte aging accompanied with the failure of fertilization [4], increased susceptibility to be activated [5], zona pellucida (ZP) hardening [6] and abnormal development of embryos [7]. However, the most important change during oocyte aging was the decrease of maturation-promoting factor (MPF) activity [5] and Kikuchi et al (2000) found that caffeine could maintain higher activity of MPF and inhibit oocyte aging in pig [8, 9]. However, evaluation of developmental competence of aged oocytes inhibited by caffeine was not completed systematically.

Caffeine is a central nervous system stimulant and acts through adenosine receptors and monoamine neurotransmitters. Many labs have tried to use caffeine to inhibit oocyte aging in different species. In pig, 5 mM caffeine decreased fragmentation rate after electrical stimulation, however, it could not increase blastocyst formation and the numbers of total cells in blastocysts. When somatic cell nuclei were injected into aged oocytes treated by caffeine, it promoted nuclear remodeling and could not prevent abnormal development of cloned embryos caused by oocyte aging [10]. Our lab found that caffeine could restore centrosome integrity and maintain spindle cytoskeleton in aged porcine oocytes [11]. In ovine, caffeine not only prevented age-related changes, such as the decline in MPF and MAPK activities [12], but also increased blastocyst formation rates of both somatic cell nuclear transfer (SCNT) embryos and Intracytoplasmic sperm injection (ICSI) embryos, reduced polyspermy and increased the glutathione after aged oocytes treated by caffeine [13, 14]. In mouse, caffeine prevented the reduced function of IP(3)R1 and live offspring from aged oocytes could be obtained after they were treated by caffeine [3, 15]. In golden hamster, caffeine delayed spontaneous oocyte parthenogenetic activation for at least 5 hr, however, it accelerated the ZP hardening [16].

Controlling oocyte aging is very important not only for health reproduction, but also for extending the time for oocyte manipulation. For example, more and more children are produced by *in vitro fertilization* (IVF). The average fertilization rate after standard IVF is about 60-70% [17], which required to re-inseminate aged unfertilized oocytes failing in IVF cycles by ICSI. Studies showed that only 38% oocytes aged for 1 day that could be fertilized using ICSI and this rate was much lower than that of fresh oocytes after standard ICSI (64.2%). Even if these aged oocytes could form pronucleus (PN), their implantation and developmental potential were very low [18]. In agricultural fields, animal embryo engineering need more oocyte to make *in vitro* produced embryos, such as IVF embryos or cloned embryo. However, the time for oocyte manipulation is limited. So controlling or delaying oocyte aging might provide more time for these procedures.

It was reported that mouse oocytes lost their developmental potential by 14-18 hr after ovulation and caffeine extended this time to 16-22 hr [3]. However, how and what aspects caffeine inhibited oocyte aging had not been investigated systematically. It showed that spindle analysis, distribution of cortical granules (CGs), zona pellucida (ZP) hardening and PN formation after parthenogenetic activation or fertilization were used to assess oocyte quality [1]. In this study, we used these indicators to evaluate aged oocytes treated by caffeine for 24 hr (an ultimate time for oocyte aging in mouse). This study would provide more evidence to show the safety of using caffeine in both human assisted reproduction technologies (ART) and agricultural animal embryo engineering.

## RESULTS

### Caffeine inhibited the separation of cumulus cells from oocytes during aging

**Figure 1:**
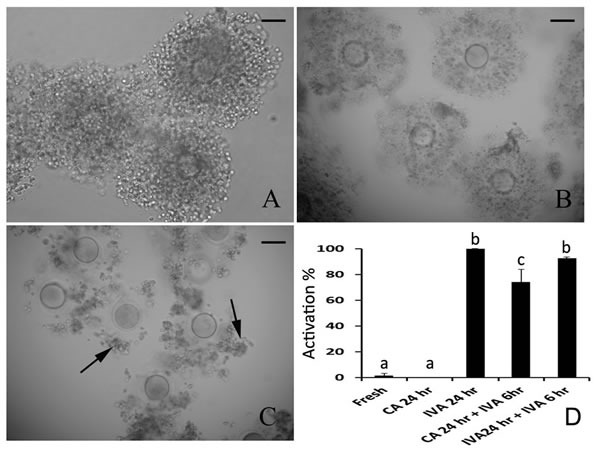
The effects of caffeine on the cumulus cells attaching and parthenogenetic activation during oocyte aging **A**. Fresh oocytes with full cumulus cells attached. **B**. Caffeine-treated aged oocytes for 24 hr with most cumulus cells attached. **C**. Oocytes aged for 24 hr *in vitro* with few cumulus cells attached. **D**. The activation of fresh oocytes, caffeine treated oocytes and oocytes aged *in vitro* after parthenogenetic activation. All graphs show mean ± s.e.m. Abbreviations used in this and all subsequent figures: CA, caffeine treated; IVA, *in vitro* aging. a-c: Values without a common letter in their superscripts differ significantly (*P* < 0.05). The black arrows indicate the unattached cumulus cells in aged COCs. Bar, 80 μm.

Cumulus cells accelerated aging of mouse oocytes as we previously reported [19]. To avoid the interference of cumulus cells on oocyte aging, we observed whether caffeine would keep the attachment of cumulus cells on oocytes during aging. It was shown that all cumulus cells were attached in fresh COCs (Figure 1A) and most cumulus cells were attached in COCs treated by caffeine (Figure 1B). However, most cumulus cells were separated in COCs aged for 24 hr (Figure 1C). So we ruled out the possibility that the separation of cumulus cells from oocytes contributed to the aging inhibition by caffeine.

### Effects of caffeine on the activation of aged oocytes treated by caffeine

It has been proved that caffeine could inhibit oocyte aging effectively in mouse [3]. We found that aged oocytes treated by caffeine for 24 hr were hardly activated by weak stimulation like fresh oocytes. However, if these treated oocytes were aged for further 6 hr in CZB, there were 74.2% treated aged oocytes could be activated by weak stimulation again and this activation percentage was very close to that of oocytes aged for 24 hr and 30 hr (Figure 1D). So we concluded that aged oocytes treated by caffeine still could have competence to be activated.

### Caffeine maintained the spindle morphology and changed CGs distribution of aged oocytes

**Figure 2:**
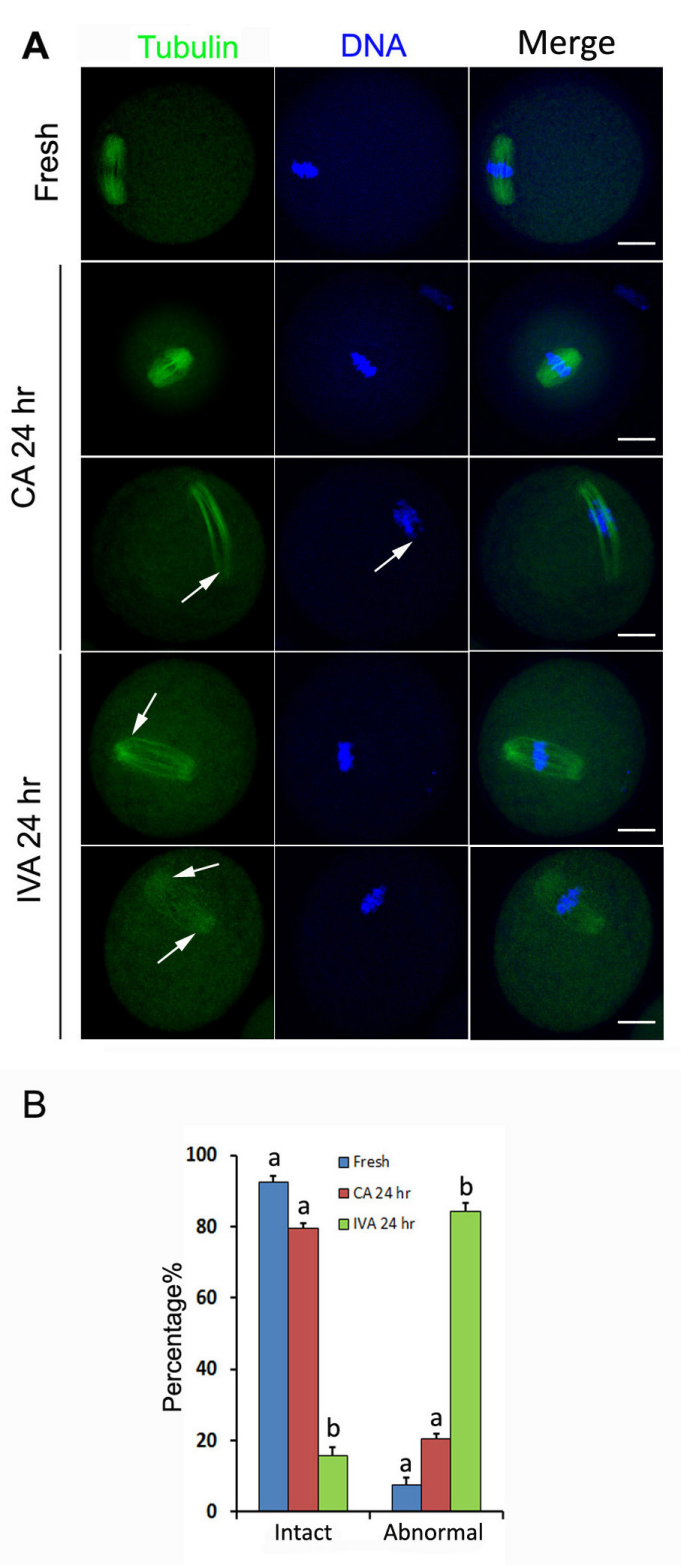
The effects of caffeine on the spindle morphology during oocyte aging **A**. Spindle morphology of fresh oocytes, caffeine-treated oocytes and oocytes aged *in vitro*. **B**. Percentage of oocytes with intact and abnormal spindles in fresh oocytes, caffeine-treated oocytes and oocytes aged *in vitro*. All graphs show mean ± s.e.m. Abbreviations used in this and all subsequent figures: CA, caffeine treated; IVA, *in vitro* aging. a-c: Values without a common letter in their superscripts differ significantly (*P* < 0.05). Spindle (green) and chromosomes (blue). The white arrows indicate the spindle defects and chromosome misalignment. Bar, 20 μm.

Intact spindles displayed bipolar spindles with focused poles in oocytes. There are 92.4% oocytes showed the intact spindle morphology in fresh oocytes. However, if oocytes were aged for 24 hr *in vitro*, microtubules become gradually lost from the spindle, with preferential loss in the central spindle area near the chromosomes. Astral fibers radiated out from the polar centrosomes into the cytoplasm and astral microtubules in the cytoplasm became gradually depolymerized. There were only 15.7% oocytes showed intact spindle morphology in aged oocytes (Figure 2A). However, 79.5% aged oocytes showed intact spindle morphology after treated by caffeine for 24 hr. So spindle morphology could be recovered by caffeine in aged oocytes (Figure 2B).

**Figure 3:**
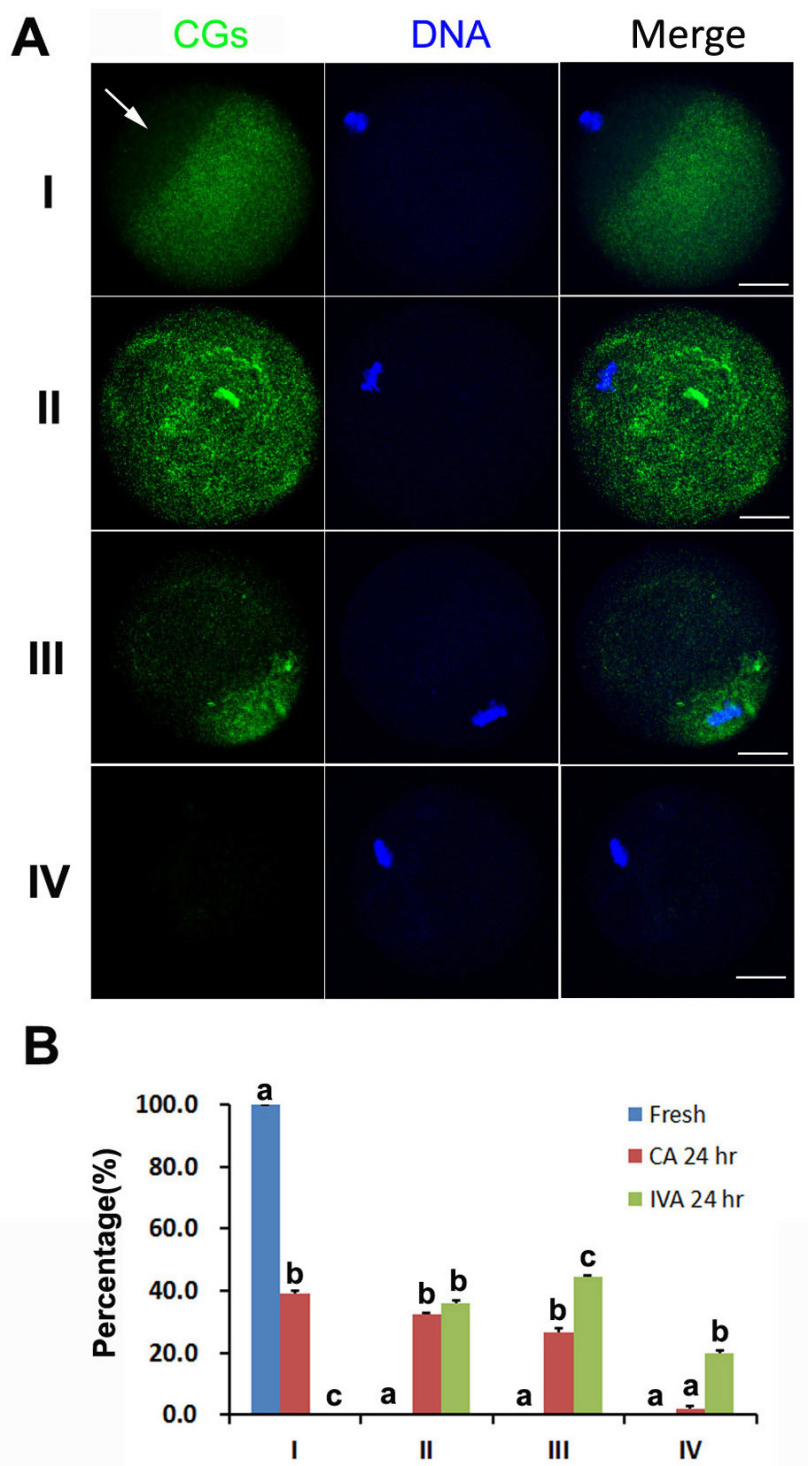
The effects of caffeine on the CGs distribution during oocyte aging **A**. Four types of CGs distribution are shown in different oocytes. **B**. Percentage of oocytes with different CGs distribution in fresh oocytes, caffeine-treated oocytes and oocytes aged *in vitro*. All graphs show mean ± s.e.m. Abbreviations used in this and all subsequent figures: CA, caffeine treated; IVA, *in vitro* aging. a-c: Values without a common letter in their superscripts differ significantly (*P* < 0.05). CGs (green) and chromosomes (blue). The white arrow indicates CG free domain in fresh oocyte. Bar, 20 μm.

As we knew, CGs were released into the perivitelline space (PVS) and combined with ZP when oocytes were fertilized by sperm, which was called as cortical reaction. During oocyte aging, we found four types of CGs distribution in the oocytes. Type I: cortical granules were densely populated in a line just beneath the oolemma, with typical CG-free domains. Type II: CGs moved to all the cortex of oocytes without CG-free domains. Type III: CGs moved to CG-free domains near the chromosomes with partial exocytosis. Type IV: full exocytosis without CGs under the oolemma (Figure 3A). All oocytes showed Type I in fresh oocytes. If oocyte were aged for 24 hr, CGs distribution showed three kinds of types: Type II (36.1%), Type III (44.3%) and Type IV (19.6%). However, caffeine could inhibit partial exocytosis if oocytes were treated by caffeine for 24 hr. Very few treated oocytes (1.9%) underwent full exocytosis and 39.0% treated oocytes could maintain CGs distribution like fresh oocytes (Figure 3B). These findings indicated that caffeine could maintain spindle morphology and inhibit partial exocytosis in aged oocytes.

### Caffeine could not inhibit ZP hardening of aged oocytes

**Figure 4:**
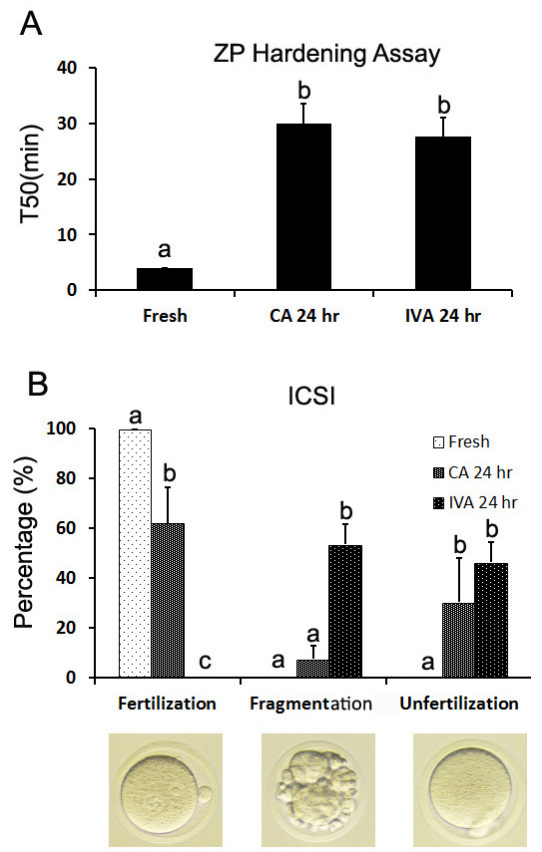
The effects of caffeine on the ZP hardening and fertilization by ICSI during oocyte aging **A**. Changes in chymotrypsin digestion time of ZP (T50 is the time at which 50% of the ZPs per group were completely digested) of fresh oocytes, caffeine-treated oocytes and oocytes aged *in vitro*. **B**. The fertilization of fresh oocytes, caffeine treated oocytes and oocytes aged *in vitro* after Intracytoplasmic sperm injection (ICSI). Abbreviations used in this and all subsequent figures: CA, caffeine treated; IVA, *in vitro* aging. a-c: Values without a common letter in their superscripts differ significantly (*P* < 0.05).

The most reason of aged oocytes failed to be fertilized was that ZP became harden in aged oocytes. We found that caffeine could inhibit CGs release partially, so we tested ZP harden by the ZP hardening assay. The results showed that the half-time (T50) for chymotrypsin-mediated dissolution of the ZP increased significantly in the ZP from both aged oocyte with or without caffeine treatment (Figure 4A), which suggested that caffeine could not inhibit CGs release completely and partial CGs were enough to induce cortical reaction and make ZP harden.

### Caffeine increased the fertilization rate of aged oocytes and decreased fragmentation by intracytoplasmic sperm injection

Aged oocytes were hardly fertilized by in vitro fertilization (IVF) because of hardened ZP. So we used ICSI as methods to test the fertilization competence of aged oocytes. We found that oocytes aged for 24 hr lost their fertilization competence. None pronucleus (PN) was formed in aged oocytes and 53.6% oocytes became fragmented. However, there were 62.2% aged oocytes treated by caffeine were fertilized normally with PN formation and only 7.5% treated oocytes became fragmentation (Figure 4B).

## DISCUSSION

Kikuchi et al. (2000) found that maturation/M-phase promoting factor (MPF) was a regulator of aging in porcine oocytes and our previous studies proved that cumulus cells accelerated oocyte aging by regulating the activity of MPF in mouse oocyte [9, 19]. Smythe et al (1992) found that caffeine could maintain higher MPF activity by inhibiting Myt1/Wee1 activity [20]. Therefore, caffeine was used to suppress Myt1/Wee1 kinase in aged oocytes and increase MPF activity, which induced low susceptibility to activating stimuli and a lower percentage of fragmentation in aged oocytes. It has been reported that caffeine was used to inhibit oocyte aging in pig, ovine, mouse and golden hamster [3, 10–12, 14, 16]. However, the safety of caffeine used to delay oocyte aging has not been investigated systematically. In this study, we used mouse oocytes as model to evaluate the safety of caffeine to inhibit oocyte aging and developmental competence of aged oocytes treated by caffeine. We employed spindle morphology analysis, distribution of CGs, ZP hardening and pronucleus (PN) formation after parthenogenetic activation or fertilization to assess their quality.

There are several morphological, cellular and molecular predictors of oocytes to evaluate the oocyte quality, including of cumulus-oocyte complex morphology, spindle morphology analysis, distribution of CGs, and developmental potential [1]. Our previous studies has proved that cumulus cells accelerated the aging progression of both *in vivo*-matured and *in vitro*-matured mouse oocytes. Further studies found that soluble Fas ligand (sFasL) secreted by cumulus cells could activate Fas on the oocyte by increasing reactive oxygen species (ROS) and glucose metabolism in cumulus cells prevented oocyte aging by producing pyruvate and NADPH through glycolysis and pentose phosphate pathway (PPP) [21–23]. To exclude the interrelationship between cumulus cells and caffeine, we found that caffeine prevented cumulus cells from separating in COCs during oocyte aging *in vitro* (Figure 1B). So caffeine inhibited oocyte aging by MPF activity without disturbance by cumulus cells. Spindle analysis is a good criterion to assess oocyte quality [24]. An intact spindle is necessary for accurate chromosome segregation, thus ensuring normal embryo development. In aged oocytes, spindles became shorter, smaller, would be bi- or multipolar. Some microtubules radiated towards the cell periphery and formed additional microtubule asters in the cytoplasm. Some centrosome structure lost at the meiotic poles [25]. We previously reported that centrosomes were absent and spindles became abnormal and disorganized in porcine oocytes aged for 48 hr, however, caffeine prevented these changes and restored centrosome integrity in the meiotic spindle poles and displayed similar meiotic spindles as those seen in fresh oocytes [11]. Our data showed that most of aged oocytes showed intact spindle morphology after treated by caffeine for 24 hr in mouse, which provided a precondition for chromosome segregation in aged oocytes (Figure 2).

Cortical granules (CGs) distribution was another important criterion to evaluate oocyte quality and they migrated to the cortex and formed a continuous layer under the oolemma [26]. In fresh oocytes, cortical reaction was triggered by sperm or artificial activation. However, it was easily triggered spontaneously without fertilization in aged oocytes. CGs become displaced and underwent partial exocytosis [27]. It was showed that caffeine accelerated CGs release in aged oocytes and normal CG distribution significantly decreased after aged for 6 hr [28]. We have reported that there were two CG distributions in aged oocytes: one was that a ring of CGs beneath the oolemma; another was that a cap of higher density CGs located above the chromosome area [29]. In our studies, we found that partial or full exocytosis occurred in oocytes aged for 24 hr. However, caffeine inhibited CGs release and full exocytosis. About 40% oocytes showed CGs distribution as seen in fresh oocytes and only 1.9% oocytes had full exocytosis (Figure 3). ZP hardening was the main reason to block fertilization, so we detected ZP hardening in oocytes treated by caffeine. Our data showed that caffeine cannot block ZP hardening. We speculate that caffeine could not inhibit CGs release completely during oocyte aging. Few CGs were released to induce cortical reaction and make ZP harden (Figure 4A). Schroeder et al. has reported that fetuin could inhibit ZP hardening and conversion of ZP2 to ZP2f during oocyte maturation *in vitro* in mouse [30], which provided an impossible way to inhibit oocyte aging using caffeine and fetuin together.

To evaluate the developmental potential of oocytes treated by caffeine, we employed weak artificial activation and ICSI to active oocytes and observe their PN formation. Fresh oocytes have low sensibility to artificial activation and are not easily activated [19]. In our studies, both fresh oocytes and aged oocytes treated by caffeine for 24 hr were hardly activated by ethanol and 6-DMAP, however, oocytes aged for 24 hr in vitro were easily activated and the activation percentage reached almost 100%. We further tested that the recoverability of oocytes treated by caffeine. Caffeine-treated oocytes were aged for 6 hr without caffeine and they were activated by ethanol and 6-DMAP. We found that 74.2% caffeine-treated oocytes were activated and formed PN, which proved that caffeine-treated oocytes could be aged again and still had potential to be activated. We also used ICSI to test the activation potential of caffeine-treated oocytes. Activation-induced fragmentation frequently occurred in aged oocytes. Our data showed that 53.6% aged oocytes became fragmentation and none of them were fertilized after sperm injection. However, oocytes treated by caffeine had a high fertilization (62.2%) and low fragmentation (7.5%) (Figure 4B). These suggested that aged oocytes treated by caffeine had a good potential to be activated.

In summary, we found that caffeine was a good oocyte aging inhibitor and most aged oocytes treated by caffeine had a developmental competence. With the development of modern ART, more matured oocytes are widely used in advanced reproductive technologies such as IVF and ICSI *in vitro*. However, the success rates of ART technologies are frequently impacted by oocyte aging, control of oocyte aging would offer a significant advantage in allowing sufficient manipulation time and selection of oocytes of the highest quality. Therefore, establishment of methods for aging control might enhance progress in ART technologies. Our data provided important information that caffeine could safely be used to control oocyte aging in related animal or clinical assisted reproductive technology.

## MATERIALS AND METHODS

### Animals and chemicals

Mice (Kunming breed) were kept in a room with 12 hr/12 hr light-dark cycles, with the dark starting from 7 PM. The mice were handled in accordance with the rules stipulated by the Animal Care and Use Committee of Huazhong Agriculture University (HZAUMO-2015-018). All chemicals were purchased from Sigma Chemical unless otherwise indicated.

### Recovery of oocytes, *in vitro* aging and caffeine treatment

To induce superovulation, female mice, 6 to 8 wk old, were given an ip treatment of 10 IU PMSG (Ningbo Hormone Product Co., Ltd., P.R. China) followed 48 hr later by ip treatment of 10 IU hCG (Ningbo Hormone Product Co., Ltd., P.R. China). Three superovulated mice in each group were killed 13 hr after hCG injection and the oviductal ampullae were broken to release the cumulus-oocyte complexes (COCs). The COCs were denuded of cumulus cells by pipetting in M2 medium (Sigma, M7167), containing 0.1% hyaluronidase (Sigma, H3506). For *in vitro* aging, the COCs were cultured in wells (25-35 oocytes per well) of a 96-well culture plate containing 200 ml of Chatot- Ziomek-Bavister (CZB) medium [31] and covered with mineral oil at 37.5°C under 5% CO_2_ in humidified air for 24 hr. For Caffeine treatments, the COCs were incubated with CZB supplemented with 5 mM caffeine (Sigma, C0750) for 24 hr.

### Oocyte activation and assessment

Oocytes were activated with ethanol and 6-DMAP in combination as described by Miao et al., [19]. Oocytes were first treated with 5% (v/v) ethanol in M2 medium for 5 min at room temperature, then washed three times and cultured in CZB containing 2 mM 6-DMAP for 6 hr. At the end of culture, oocytes were observed under a microscope for activation. Only those oocytes with one pronucleus or two pronuclei, or two cells each having a nucleus, were considered activated.

### Fluorescence and immunofluorescence microscopy

For spindle staining, oocytes were fixed in 4% (wt/vol) paraformaldehyde, washed in blocking buffer (PBS containing 0.3% BSA, 0.01% Tween-20, and 0.01% NaN3), and then permeabilized in PBS containing 0.3% BSA, 0.1% Triton X-100, and 0.01% NaN3. The oocytes were incubated in primary anti-α-tubulin antibody (Sigma, F2168) diluted 1:100 in blocking buffer. For CG staining, zonae pellucidae were removed by brief incubation in acid Tyrode's solution. The oocytes were fixed in 4% (wt/vol) paraformaldehyde in PBS, blocked in PBS containing 0.3% BSA and 100 mM glycine, and then permeabilized in PBS containing 0.1% Triton X-100. CGs were labeled in 100 μg/mL FITC-conjugated peanut agglutinin in PBS. The oocytes were washed in blocking buffer and mounted as above. Slides were scanned by using a Zeiss confocal microscope (Zeiss LSM 510 UV).

### Assay for zona pellucida hardening

The assay for zona pellucida (ZP) hardening was performed as described by Gulyas and Yuan [32] with minor modifications. Briefly, 20 cumulus-free oocytes were treated with 1 mg/ml achymotrypsin (Sigma C4129) contained in a 100 ml drop of PBS covered with mineral oil at 30°C. Oocytes were monitored every 2 min during the first 30 min of the treatment and then every 5 min until the end of the treatment (3 hr). The time at which 50% of the ZP underwent a complete dissolution (with denuded oocytes stuck on the bottom of the dish) was assessed as T50 for ZP dissolution.

### Intracytoplasmic sperm injection

Cauda epididymal sperm were collected from Kunming males into HTF medium (Millipore, MR-070-D). A single sperm head was microinjected into an MII oocyte by using a NIKON Ti-S inverted microscope equipped with a Narishige Micromanipulator system and an Eppendorf Piezo drill. Only those oocytes with two pronuclei were considered as fertilization and oocytes showed like cleavage without pronuclei were considered as fragmentation (Figure 4B).

### Data analysis

For each treatment, three replicates were run. Statistical analyses were carried out by analysis of variance. Differences between groups treated were evaluated with the Duncan multiple comparison test. Data are expressed as mean ± SEM and *P* < 0.05 is considered significant.
